# Enhancing Patient Safety in Critical Care Settings: Lessons from a Multidimensional Special Issue

**DOI:** 10.3390/healthcare14142181

**Published:** 2026-07-20

**Authors:** Meropi Mpouzika

**Affiliations:** Department of Nursing, School of Health Sciences, Cyprus University of Technology, Limassol 3036, Cyprus; meropi.mpouzika@cut.ac.cy

Patient safety has been conceptualized as a system-level outcome that depends on coordinated efforts among healthcare professionals, effective organizational processes, and strategies aimed at preventing medical errors, avoiding preventable adverse events, and protecting patients from harm [1]. This understanding of patient safety is particularly relevant to critical care settings. Intensive care units and emergency departments manage highly vulnerable patients exposed to the risks associated with severe illness, complex therapeutic interventions, and rapidly changing clinical conditions. Consequently, these settings are associated with an increased risk of preventable harm [2,3,4,5,6] due to patient complexity, the urgent nature of clinical decision-making, high workload, frequent invasive procedures, and the intensity of care delivery [7]. Within this context, ensuring safe care in critical care settings remains an ongoing challenge. The eleven articles accepted for publication in this Special Issue of *Healthcare*, *Enhancing Patient Safety in Critical Care Settings*, illustrate the multifaceted nature of patient safety in critical care and form the basis for the thematic discussion presented below. Table 1 summarizes the eleven contributions according to their theme, primary focus, study design, and clinical setting, whereas the List of Contributions contains the complete bibliographic details.

Building on these contributions, five interrelated themes provide the framework for the discussion presented below. Evidence-based practice represents a cornerstone of patient safety in critical care. Translating evidence into routine clinical practice remains a recurring challenge across several contributions included in this Special Issue. In critical care settings, the benefits of evidence-based interventions depend not only on the availability of evidence but also on the ability of healthcare professionals and organizations to implement it effectively. However, generating evidence alone does not guarantee safer care. Together, these studies demonstrate that translating evidence into routine practice requires not only robust evidence but also education, professional competencies, and organizational support [8,9,10].

This evidence-to-practice gap is reflected in the study by Papadimitriou et al. [Contribution 1], where physiotherapists acknowledge the benefits of early mobilization but report several patient-, provider-, and institutional barriers that limit its implementation. Similarly, the study on delirium management [Contribution 2] reveals that although ICU nurses demonstrate moderate-to-high levels of knowledge, routine screening and protocolized management remain suboptimal, highlighting the need for structured education and implementation strategies. Likewise, the pilot study on nurse-performed FAST examinations [Contribution 3] demonstrates how advanced nursing competencies may support rapid bedside assessment and clinical decision-making in emergency care settings. Overall, translating evidence into routine clinical practice requires not only robust evidence but also education, professional competencies, structured clinical protocols, and organizational support.

Another key dimension of patient safety highlighted in this Special Issue is the timely recognition of clinical deterioration. The study on Early Warning Scores [Contribution 4] identifies workforce and organizational barriers that limit the effective use of these systems despite the potential to facilitate early intervention. These findings reinforce that early recognition systems are effective only when accompanied by adequate organizational support, training, and integration into standard clinical workflows.

A resilient and well-supported workforce represents another fundamental component of patient safety in critical care. Beyond the implementation of evidence-based clinical practices, workforce well-being, resilience, and the prevention of workplace violence remain important priorities for healthcare organizations, given their close association with the delivery of safe patient care [11,12,13,14].

The study by Bellali et al. [Contribution 5] demonstrates that self-care and self-compassion are associated with better job performance among critical care nurses, underscoring the importance of healthcare professionals’ psychological well-being. The role of resilience is further emphasized by the narrative review of resilience-building interventions [Contribution 6], which illustrates the potential benefits of such programs for nurses and the need for sustained organizational support. Workplace violence emerges as another major challenge affecting both staff well-being and patient safety. The quantitative study on emergency department nurses highlights the interplay between resilience, communication skills training, and supportive organizational environments in mitigating the adverse professional consequences of workplace violence [Contribution 7]. Complementing these findings, the qualitative study identifies a pronounced “empathy gap” between nurses, patients, relatives, and healthcare organizations, emphasizing the importance of addressing divergent expectations and strengthening organizational responses to workplace violence [Contribution 8].

Patient safety extends beyond the prevention of physical harm to encompass patients’ psychological well-being and experience of care. While efforts to improve safety have traditionally focused on preventing physical harm, increasing attention has been directed toward understanding how critically ill patients experience care and how psychological distress during intensive care unit (ICU) hospitalization may affect recovery and long-term well-being [15].

This perspective is reflected in the meta-ethnographic synthesis of ICU patients’ psychological responses, which identifies a pervasive sense of disempowerment and loss of control during critical illness. The concept of the “disempowered warrior” underscores the role of empathy, communication, and patient involvement in promoting recovery and well-being [Contribution 9]. Consistent with these findings, the scoping review of psychosocial support interventions demonstrates the potential of psychosocial and psychological interventions to reduce distress and support recovery among critically ill patients. Nevertheless, the review identifies important evidence gaps and emphasizes the limited integration of psychosocial support within current critical care guidelines [Contribution 10].

The safe use of increasingly complex therapies remains another essential aspect of patient safety in critical care. Given the complexity of contemporary therapies in critically ill patients [2,16], continuous assessment of treatment safety is essential. The contribution examining remdesivir-associated hepatotoxicity and acute kidney injury illustrates the continuing need for pharmacovigilance and careful monitoring of potential adverse effects, reminding clinicians that therapeutic effectiveness must always be balanced against the potential for harm [Contribution 11].

The studies included in this Special Issue also point to several priorities for future research. Future research should focus on implementation strategies that facilitate the translation of evidence into practice, the development and evaluation of workforce-support interventions, the integration of psychosocial care into routine critical care, and the incorporation of patient-centered outcomes alongside traditional safety indicators when evaluating patient safety interventions.

Taken together, the studies in this Special Issue reinforce a fundamental lesson: ensuring the safety of critically ill patients requires more than isolated interventions; it depends on the alignment of evidence, people, and systems. As critical care continues to evolve, further advances in patient safety will depend not only on scientific and technological progress, but also sustained commitment to the human and organizational dimensions of care.

## Figures and Tables

**Table 1:** Overview of the eleven contributions included in the Special Issue.

No. of Contribution	Theme	Focus	Study Design	Clinical Setting
1	Evidence-based practice	Early mobilization implementation	Cross-sectional survey	ICU
2	Evidence-based practice	Delirium management practices	Cross-sectional study	ICU
3	Evidence-based practice	Nurse-performed FAST examination	Pilot study	ED
4	Early recognition of clinical deterioration	Implementation of Early Warning Scores	Implementation study	Acute care hospitals
5	Workforce well-being and resilience	Psychosocial predictors of job performance	Cross-sectional study	ICU and ED
6	Workforce well-being and resilience	Long-term impact of resilience-building interventions	Narrative review	Multiple healthcare settings
7	Workforce well-being and resilience	Workplace violence, resilience and turnover intention	Cross-sectional study	ED
8	Workforce well-being and resilience	Workplace violence and the empathy gap	Qualitative study	ED
9	Patient experience	Psychological distress, disempowerment and the “Disempowered Warrior” experience	Meta-ethnography	ICU
10	Patient experience	Psychosocial support interventions for psychological well-being	Scoping review	ICU
11	Treatment safety	Remdesivir-associated hepatotoxicity and acute kidney injury	Multicenter retrospective cohort study	Acute care hospitals

Abbreviations: ED, Emergency Department; FAST, Focused Assessment with Sonography for Trauma; ICU, Intensive Care Unit.
